# Supplementary material for: Lacticaseibacillus casei IB1 Alleviates DSS-Induced Inflammatory Bowel Disease by Regulating the Microbiota and Restoring the Intestinal Epithelial Barrier
Source: Microorganisms. 2024 Jul 6;12(7):1379. doi: 10.3390/microorganisms12071379 (PMC11278699; doi:10.3390/microorganisms12071379)
Supplement: Supplementary file 1 [file microorganisms-12-01379-s001.zip › microorganisms-2995780-supplementary.pdf]

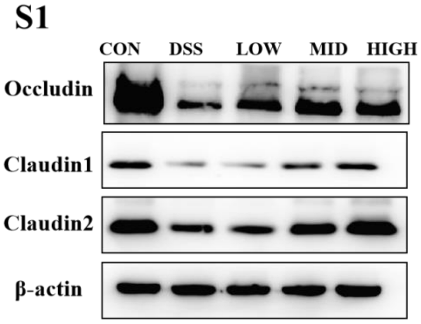

Figure S1. Western blot analysis of Occludin, Claudin1 and Claudin2 levels in the colon.

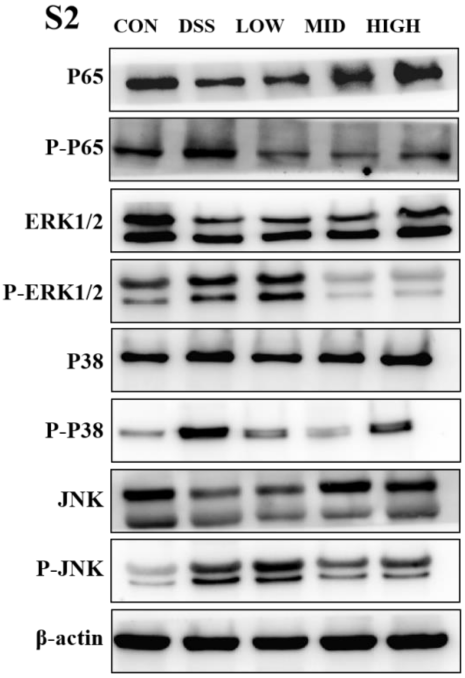

Figure S2. Western blot analysis of P65, P-P65, ERK1/2, P-ERK1/2, P38, P-P38, JNK and P-JNK levels in the colon.
